# Transforming Informed Consent Generation Using Large Language Models: Mixed Methods Study

**DOI:** 10.2196/68139

**Published:** 2025-02-13

**Authors:** Qiming Shi, Katherine Luzuriaga, Jeroan J Allison, Asil Oztekin, Jamie M Faro, Joy L Lee, Nathaniel Hafer, Margaret McManus, Adrian H Zai

**Affiliations:** 1 Center for Clinical and Translational Science, University of Massachusetts Chan Medical School, 55 N Lake Ave, Worcester, MA, 01655, United States, 1 508-856-1952; 2 Department of Population and Quantitative Health Sciences, University of Massachusetts Chan Medical School, Worcester, MA, United States; 3 Manning School of Business, University of Massachusetts Lowell, Lowell, MA, United States

**Keywords:** informed consent form, ICF, large language models, LLMs, clinical trials, readability, health informatics, artificial intelligence, AI, AI in health care

## Abstract

**Background:**

Informed consent forms (ICFs) for clinical trials have become increasingly complex, often hindering participant comprehension and engagement due to legal jargon and lengthy content. The recent advances in large language models (LLMs) present an opportunity to streamline the ICF creation process while improving readability, understandability, and actionability.

**Objectives:**

This study aims to evaluate the performance of the Mistral 8x22B LLM in generating ICFs with improved readability, understandability, and actionability. Specifically, we evaluate the model’s effectiveness in generating ICFs that are readable, understandable, and actionable while maintaining the accuracy and completeness.

**Methods:**

We processed 4 clinical trial protocols from the institutional review board of UMass Chan Medical School using the Mistral 8x22B model to generate key information sections of ICFs. A multidisciplinary team of 8 evaluators, including clinical researchers and health informaticians, assessed the generated ICFs against human-generated counterparts for completeness, accuracy, readability, understandability, and actionability. Readability, Understandability, and Actionability of Key Information indicators, which include 18 binary-scored items, were used to evaluate these aspects, with higher scores indicating greater accessibility, comprehensibility, and actionability of the information. Statistical analysis, including Wilcoxon rank sum tests and intraclass correlation coefficient calculations, was used to compare outputs.

**Results:**

LLM-generated ICFs demonstrated comparable performance to human-generated versions across key sections, with no significant differences in accuracy and completeness (*P*>.10). The LLM outperformed human-generated ICFs in readability (Readability, Understandability, and Actionability of Key Information score of 76.39% vs 66.67%; Flesch-Kincaid grade level of 7.95 vs 8.38) and understandability (90.63% vs 67.19%; *P*=.02). The LLM-generated content achieved a perfect score in actionability compared with the human-generated version (100% vs 0%; *P*<.001). Intraclass correlation coefficient for evaluator consistency was high at 0.83 (95% CI 0.64-1.03), indicating good reliability across assessments.

**Conclusions:**

The Mistral 8x22B LLM showed promising capabilities in enhancing the readability, understandability, and actionability of ICFs without sacrificing accuracy or completeness. LLMs present a scalable, efficient solution for ICF generation, potentially enhancing participant comprehension and consent in clinical trials.

## Introduction

Ethical codes and regulations have been established globally to guide researchers in conducting studies involving human subjects. In the United States, the Belmont Report and the Common Rule are key frameworks for ensuring ethical research practices. The Common Rule, formally known as the “Basic Department of Health and Human Services (HHS) Policy for the Protection of Human Research Subjects,” requires that participants receive comprehensive information about the study’s purpose, allowing them to make informed and autonomous decisions about their participation [1]. This process of obtaining informed consent is fundamental to responsible conduct in research involving human subjects [2]. However, in recent years, the inclusion of mandatory scientific content, legal jargon, and increasing length has turned the informed consent form (ICF) into a barrier to study participation [3,4].

Although many institutional review boards (IRBs) require investigators to develop documents written at the eighth-grade reading level [5], research has found that research ICFs are frequently written at reading grade levels that far exceed readers’ abilities [2,5-9].

In response to the increasing complexity and length of informed consent documentation, the Health and Human Services Office for Human Research Protections added a new requirement to the 2018 Common Rule, stipulating that ICFs must begin with “a concise and focused presentation of the key information that is most likely to assist prospective subjects in understanding the reasons why one might or might not want to participate in the research” and that it “must be organized and presented in a way that facilitates comprehension” [1]. The Secretary’s Advisory Committee on Human Research Protections has recommended conducting empirical research to guide the writing of the new key information section in light of the new consent requirement, ensuring that its goals are effectively met [10].

With the advancement of large language models (LLMs), a possible solution to improving ICF has emerged. LLMs show significant potential in health informatics, including tasks such as name entity extraction [11], patient trial matching [12,13], biomedical reasoning and classification [14], prediction of admissions [15], automation of administrative tasks [16], and so forth. Studies have also shown that LLMs can effectively enhance the documentation of risks, benefits, and alternatives for common surgical procedures [17]. The integration of LLMs in clinical workflows could significantly reduce administrative burden by automating labor-intensive tasks such as ICF creation. However, for successful implementation, models must not only improve readability and actionability but also align with current regulatory requirements and ethical guidelines.

The ability for LLMs to generate a complex clinical trial ICFs from a research protocol remains unexplored. This paper aims to evaluate the performance of the Mistral 8x22B LLM in generating the key information sections of ICFs with improved readability, understandability, and actionability. Specifically, our objectives are to assess the model’s effectiveness in producing ICFs that meet readability standards, enhance understandability, and support actionable content while maintaining accuracy and completeness. Furthermore, we hypothesize that LLM-generated ICFs outperform human-generated counterparts in readability, understandability, and actionability, without compromising on the accuracy or completeness of information.

## Methods

### Study Design

We sourced 4 research protocols from the UMass Chan IRB, along with their corresponding ICFs. These protocols were then processed by our LLM model to generate artificial intelligence (AI)–generated ICFs, resulting in a total of 8 ICFs—4 human-generated and 4 AI-generated. Each research protocol, along with its respective human and AI-generated ICFs, was randomly assigned to evaluators for assessment. We had 8 evaluators in total, ensuring that each protocol set was reviewed twice by 2 different evaluators. A multidisciplinary team of 8 evaluators, including health informaticians, clinical researchers, and physicians, was assembled to review the outputs. Importantly, the evaluators were not investigators in the clinical trials whose ICFs they assessed and were not directly affiliated with the specific studies under review. Care was also taken to ensure that the evaluators and the investigators for each protocol were from different departments within the institution. Furthermore, none of the evaluators were members of the IRB that reviewed and approved the protocols. These measures were implemented to minimize potential bias and ensure objective evaluation.

Each protocol set was evaluated by 2 different reviewers, ensuring comprehensive assessment. The evaluation focused on key criteria: completeness, accuracy, readability, understandability, and actionability of the generated content. To mitigate potential evaluator bias, we ensured that each protocol was randomly assigned and evaluated by multiple individuals from different disciplines. This multidisciplinary approach, combined with random assignment, reduces the risk of personal bias and ensures a more comprehensive assessment of both LLM and human-generated ICFs.

### Study Protocols

The 4 protocols included in this study were selected to ensure diversity in study design, therapeutic areas, and patient populations. This approach was aimed at evaluating the generalizability of LLM-generated ICFs across varied research contexts. Table 1 summarizes the key attributes of the protocols.

**Table 1.** Summary of study protocols.

Study title	Study type	Domain
Kangaroo Mother Care Study	Qualitative study	Neonatology
Characterization of Oral Microbiome in Patients With Viral Respiratory Illness	Observational cohort study	Infectious Diseases, Microbiome
RADx Tech COVID-19 Test Us Study	Platform trial	Infectious Diseases, Diagnostics
Healthy at Home Pilot	Pilot feasibility trial	Pulmonology, Digital Health

### LLM Model

We chose the Mistral 8x22B model, the latest offering from Mistral [18], for several compelling reasons:

1. Large Context Window: With a 64K token context window, this model can manage extensive research protocols. It is ideal for accurately recalling information from large documents such as clinical trial research protocols.
2. Multilingual Fluency: The Mistral 8x22B excels in multiple languages, aligning with our objective of using LLMs to create ICFs that ensure fair recruitment and serve underrepresented populations. Producing ICFs in various languages, such as Spanish, is highly advantageous.
3. Open-Source License: The Mistral 8x22B is available under an Apache 2.0 open-source license [19], allowing unrestricted deployment. This flexibility is beneficial as we roll out the final product.

### ICF Key Information Section Integration

We downloaded ICF templates from various institutions, including the University of California San Francisco, Yale University, Duke University, New York University, the University of Pennsylvania, Johns Hopkins University, Partners HealthCare, Stanford University, Vanderbilt University, and the UMass Chan Medical School. We then consolidated the key information section instructions provided by these institutions into a comprehensive format. To reinforce this format, we made modifications following the Readability, Understandability, and Actionability of Key Information (RUAKI) indicator, ensuring that our consolidated key information sections create more accessible information. The final version of this format serves as the key information section instruction input for the LLM model.

### Prompt Engineering

To create the ICF key information content, we used Mistral artificial intelligence (AI) in conjunction with prompt engineering guidance developed by the Research Informatics Core as part of a human-in-the-loop process. This team included the chief research information officer, 2 clinical data scientists, and an IRB officer. The prompt creation followed a backward design instructional approach [20]. The consolidated key information section instructions were used to design the prompts. The data scientists then took this guidance and crafted prompts to align with these instructions.

We used a Least-to-Most approach to guide the AI through the process of creating the consent forms. This step-by-step approach ensured that the AI received small, manageable instructions at each stage, helping it produce more accurate and reliable outputs. By breaking down tasks into smaller steps rather than overwhelming the AI with multiple instructions at once, we reduced confusion and enhanced the quality of the AI-generated forms. After designing and developing the key information section, the output was rated and reviewed by the Research Informatics Core team. Based on their feedback, the prompts were edited to enhance the model’s performance.

We started by creating the chatbot prompt detailed in Supplementary 1 in Multimedia Appendix 1 to extract relevant information for each key section from the research protocol. Next, as described in Supplementary 2 in Multimedia Appendix 1, we refined the output using RUAKI indicators. In Supplementary 3 in Multimedia Appendix 1, we adjusted the content to achieve Flesch-Kincaid grade levels below 8. Finally, in Supplementary 4 in Multimedia Appendix 1, we formatted the output to align with our preferred forms, again guided by RUAKI indicators.

### Measurements of Accuracy and Completeness

To evaluate accuracy and completeness, we developed a scoring system based on recommendations from LeapFrog (VTech Group), The Joint Commission, the American College of Surgeons, and relevant available literature (Multimedia Appendix 2) [21-24]. Key information sections, including study purpose, duration and procedures, risks and discomforts, benefits, and alternatives, were assessed as complete, incomplete, absent, or incorrect, with corresponding scores of 3, 2, 1, and 0, respectively.

### Measurements of Readability, Understandability, and Actionability

We used the RUAKI indicator to evaluate readability, understandability, and actionability presented in ICFs key information section [25]. This indicator consists of 18 items, each assessed with a binary rating of “yes” (scored as 1) or “no” (scored as 0) (Table 2). To determine the final score, we sum the number of “yes” responses, divide by the total number of items (18), and multiply by 100 to yield a percentage score. The section score for readability, understandability, and actionability was derived by dividing the final score of each section by the total number of relevant items. A higher percentage indicates that the key information is more accessible, comprehensible, and actionable.

**Table 2.** Readability, understandability, and actionability of key information evaluation criteria.

Category and item number		Description	Rating
Readability			
	1	Active voice: uses active verbs (eg, will use) rather than passive verbs (eg, will be used) all or most of the time, more than 90% of the time.	Yes=1/No=0
	2	Word choice: avoids scientific jargon (eg, hypertension). Uses words readers are familiar with (eg, high blood pressure) all or most of the time, more than 90% of the time.	Yes=1/No=0
	3	Topic definition: provides a definition of the main disease or topic the study is about.	Yes=1/No=0
	4	Numbers: avoids mathematical calculations including comparison of numeric probability of risk.	Yes=1/No=0
	5	Eighth grade or below: reading grade level calculated in Microsoft Word is Flesch-Kincaid grade level 8.9 or below.	Yes=1/No=0
	6	Headers: sections or chunks of information are labeled with headers. Headers clearly describe sections so that readers can scan and find information.	Yes=1/No=0
	7	Font type and size: font type or style is easy to read. Font size is at least 11‐12 point.	Yes=1/No=0
	8	White space: uses bulleted or numbered lists to increase white space on the page.	Yes=1/No=0
	9	Image: contains at least 1 image that is related to the topic of the study. Not a logo.	Yes=1/No=0
Understandability			
	10	Purpose of the study: includes a statement that says, “the purpose of the study is…” Purpose of the study is stated, rather than implied.	Yes=1/No=0
	11	Main reason to join the study—benefits: includes description or list of potential benefits to participants or others.	Yes=1/No=0
	12	Main reasons not to join the study—risks: includes description or list of potential side effects or risks to participants.	Yes=1/No=0
	13	Information being collected: describes the information that will be collected from participants and about participants.	Yes=1/No=0
	14	Study procedures: describes what participants will need to do AND how much time it will take.	Yes=1/No=0
	15	Study is research: includes a statement that says, “study is research” or “research study” not just consenting to treatment.	Yes=1/No=0
	16	Participation is voluntary: states that participation is voluntary and that participants have a choice to be in the study or not.	Yes=1/No=0
	17	Costs and compensation: describes any financial payments (or costs) to study participants.	Yes=1/No=0
Actionability			
	18	Consent process: describes the process by which the reader gives his or her consent by signing a document, verbal agreement, via computer, or other.	Yes=1/No=0

### Statistical Analysis

We reported mean accuracy and completeness scores for human-generated and LLM-generated ICF key information sections. We compared the mean accuracy and completeness scores of human-generated and LLM-generated ICF key information sections using Wilcoxon rank sum tests. Furthermore, we compared the RUAKI indicators between the 2 groups using the Wilcoxon rank sum test. Moreover, we measured the intraclass correlation coefficient (ICC) to assess the consistency among raters. An ICC below 0.5 indicates low reliability, between 0.5 and 0.74 indicates moderate reliability, from 0.75 to 0.9 suggests good reliability, and above 0.9 signifies excellent reliability [26].

### Ethical Considerations

This study qualifies as nonhuman subjects research under applicable institutional and regulatory guidelines, as it exclusively involved evaluators who are also coauthors of this work. No external participants were involved, and no identifiable private information was collected, analyzed, or shared. Consequently, this work did not require review or approval from an IRB.

## Results

The accuracy and completeness of the LLM- and human-generated outputs were comparable across key sections of the ICFs (Table 3). Both the LLM and human outputs achieved similar scores for conveying the study purpose (2.88 vs 2.63), with no significant difference (*P*=.16). For the duration and procedures, the scores were also close (2.5 vs 2.38), with no statistically significant difference (*P*=.56). The LLM slightly outperformed the human output in explaining the risks and discomforts (2.63 vs 2.38), but again, this difference was not statistically significant (*P*=.32). In terms of benefits, the LLM achieved a perfect score of 3.0 compared with the human output’s 2.57, although this difference approached but did not reach statistical significance (*P*=.10). Both the LLM and human outputs were identical in discussing alternatives, scoring 2.75 (*P*≥.99). For the overall impression, the LLM scored 2.63 compared with the human output’s 2.31, with no statistically significant difference (*P*=.32). Overall, both outputs displayed comparable levels of performance across these key sections.

**Table 3.** Mean accuracy and completeness scores for human and large language model evaluations across key informed consent sections.

	LLM output, mean score (SD)	Human output, mean score (SD)	Wilcoxon rank sum tests, *P* value
Study purpose	2.88 (0.35)	2.63 (0.52)	.16
Duration and procedures	2.5 (0.53)	2.38 (0.52)	.56
Risks and discomforts	2.63 (0.52)	2.38 (0.52)	.32
Benefits	3 (0)	2.57 (0.79)	.10
Alternatives	2.75 (0.46)	2.75 (0.46)	≥.99
Overall impression	2.63 (0.52)	2.31 (0.59)	.32

**Figure 1.**
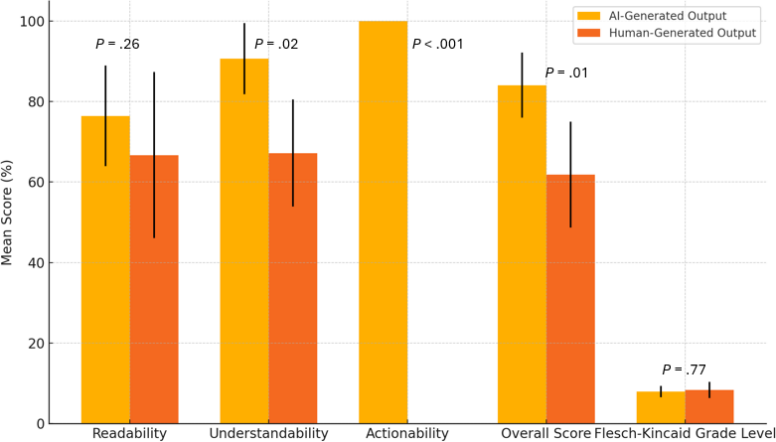
Comparison of AI- and human-generated informed consent form performance: mean Readability, Understandability, and Actionability of Key Information scores with CIs and Wilcoxon signed rank test *P* values for readability, understandability, actionability, and Flesch-Kincaid grade level. AI: artificial intelligence.

The comparison of Mean RUAKI scores for ICF key information generated by LLMs versus human output reveals that the LLM consistently outperforms human-generated content in critical areas (Figure 1). Although both the LLM and human outputs achieved relatively high readability scores, with the LLM slightly ahead (76.39% vs 66.67%), this difference approached but did not reach statistical significance (*P*=.26). The LLM demonstrated significantly better understandability, scoring 90.63% compared with the human score of 67.19%, with a statistically significant *P* value of .015. Moreover, the LLM consistently included an actionable next step at the end of the document, a crucial element that the human output failed to provide, as evidenced by the LLM’s perfect actionability score of 100% compared with 0% for the human output. Overall, the LLM’s content achieved a significantly higher combined score (84.03% vs 61.82%), with a statistically significant *P* value of .008, demonstrating that LLM-generated text is generally more effective in producing ICF key information sections that are not only easier to read but also more understandable and actionable for participants.

While both the LLM- and human-generated ICFs exhibited similar grade levels, the LLM generated content at a slightly lower grade level (7.95 vs 8.375), indicating that it is easier to read and better aligned with the recommended reading level for general audiences. However, this difference was not statistically significant (*P*=.77), suggesting comparable readability between the two. Nonetheless, the LLM’s content remains closer to the target readability level, providing a subtle advantage in ensuring accessibility for a wider audience.

The ICC score for the average ratings across the raters was found to be 0.83 (95% CI 0.64-1.03). According to the general interpretation guidelines for ICC values, this score indicates good reliability.

## Discussion

### Principal Results

This study evaluated the performance of the Mistral 8x22B LLM in generating key information sections for ICFs in clinical trials. The comparison between LLM-generated content and human-generated ICFs revealed that LLMs demonstrate considerable potential for improving the efficiency, readability, and actionability of ICFs, while maintaining comparable accuracy and completeness across most assessed categories.

#### Accuracy and Completeness

The LLM-generated ICFs achieved comparable performance to human-generated content across most areas. Both LLM and human outputs were similar in conveying the purpose of the study. They also performed equally well in describing the duration and procedures. While the human-generated content slightly outperformed the LLM in discussing alternatives, the LLM performed better in explaining the benefits. The overall impression score favored the LLM slightly. These results suggest that while the LLM performs similarly to humans in most areas, further refinement in prompt engineering may be required to improve its performance in more complex sections, such as alternatives. With additional fine-tuning, LLMs could potentially match or exceed the quality of human-generated ICFs across all categories.

#### Readability

The LLM outperformed human-generated ICFs in readability, as demonstrated by higher RUAKI scores. Both LLM- and human-generated ICFs exhibited good readability according to the Flesch-Kincaid grade level, but the LLM achieved a lower average grade level, reflecting superior readability and closer alignment with institutional requirements for eighth-grade reading level content. This highlights the LLM’s strength in tackling one of the primary challenges of ICF creation: producing documents that are both comprehensive and easily understood by a general audience. Given that many ICFs often exceed the recommended reading level, the LLM’s consistent ability to generate readable content is a significant advantage, ensuring accessibility without sacrificing detail.

#### Understandability and Actionability

The LLM significantly outperformed human-generated ICFs in both understandability and actionability, as reflected in the higher RUAKI scores. The LLM’s output was not only more comprehensible but also consistently included actionable next steps, a critical component that was often missing from the human-generated content. The perfect actionability score of the LLM-generated ICFs suggests that these models can enhance participant comprehension and facilitate informed decision-making. These findings demonstrate the potential of LLMs to create ICFs that are not only easier to read but also more effective in guiding participants through the consent process.

#### Rater Consistency

The ICC of 0.83 indicates a very high level of agreement among raters, reflecting the reliability of the evaluation process. The narrow CI further supports the robustness of these ratings, ensuring the consistency and validity of the results across different protocols.

### Lessons Learned

#### Lesson 1: The Importance of Precise Temperature Settings

For all LLMs, the model temperature parameter controls the diversity of responses. A higher temperature, such as 0.8, produces more varied answers, while a lower temperature, such as 0.2, results in more focused and deterministic outputs. In our experiments, we found that setting the temperature to 0 was the most effective choice for this task. Temperatures introduce a level of randomness that can lead to hallucinations—unwanted deviations from the source material. Since our goal is to extract information directly from the research protocol and treat it as the sole source of truth, it is essential to minimize any creative output from the LLM. Although a temperature of 0.2 is already fairly focused [27,28], the need for absolute accuracy in clinical trial documents led us to set the temperature to 0, ensuring that the content remains strictly aligned with the provided data.

#### Lesson 2: Addressing Readability Challenges With Cross-Model Few-Shot Prompting

It was observed that Mistral struggled to generate content at a Flesch-Kincaid grade level of 8 when prompted directly with instructions such as, “The content should meet literacy standards, specifically an 8th-grade reading level or lower.” To overcome this challenge, unlike the other zero-shot prompts used in this project, additional prompts and a technique known as “few-shot training” [29] were introduced. This involved providing the model with examples of text at both below 8 and above 9 Flesch-Kincaid grade levels, helping to guide the model in producing content at the desired reading level. These examples, generated using ChatGPT 4, were incorporated (as shown in Supplementary 3 in Multimedia Appendix 1) enabling Mistral to produce content more consistently at the desired grade level 8 or below. This approach, known as cross-model few-shot prompting, involves using one model to generate examples (or “shots”) that are subsequently fed into another model to enhance its performance on a specific task. It is essential to apply this step after any content-editing prompts are used, as further content edits could inadvertently raise the reading level above the target. Format editing should be the final step in the process, as any subsequent prompts could alter the formatting.

#### Lesson 3: Least-to-Most Prompt Engineering

Effective prompt engineering involves breaking down tasks into manageable steps. When LLMs are given multiple instructions in a single prompt, they often struggle to follow all directions accurately. By adopting a Least-to-Most approach [30]—where each prompt contains a focused set of instructions and builds on the previous output—we achieved more consistent and reliable results. This is analogous to how clinical workflows are built iteratively in electronic health record (EHR) systems to ensure accuracy in decision-making. Much like building clinical templates or order sets, prompt engineering ensures that each phase of content generation is guided to avoid ambiguity, ensuring accuracy and relevance to the context of informed consent. For example, if we were building a medication alert in an EHR, breaking the alert logic into separate steps—from checking allergies to suggesting alternatives—ensures clarity and avoids overwhelming the user. Similarly, breaking down the prompt for generating ICF sections helps the LLM focus on retrieving the right information from the protocol. This method involves 2 stages: first, decomposing a complex problem into a series of simpler subproblems and then sequentially solving these subproblems, with each solution informed by the answers to the previous ones. By guiding the LLM to work incrementally, we not only enhanced its accuracy but also ensured that human oversight remained integral to the process, leading to optimal outcomes and greater control over the final product. For instance, one reviewer noted that the LLM-generated procedures were missing some procedure information. This issue likely stemmed from the lengthy and poorly structured procedure section in the original research protocol. To address this, we designed a workflow in which we first asked the LLM to summarize the study’s procedures and timeline. We then instructed the LLM to extract the necessary information from this summarized output. This step-by-step Least-to-Most approach allowed us to successfully extract the missing information and integrate it into the key information.

#### Lesson 4: Real-World Application and Integration Challenges

Implementing LLMs in clinical workflows requires more than just improving readability or accuracy—it necessitates seamless integration with existing clinical systems and processes, such as EHRs and IRB workflows. For LLMs to have a real-world impact, models need to be adaptable to diverse clinical environments and meet regulatory and ethical standards. One way to ensure this is by developing interfaces that allow researchers to fine-tune LLM outputs while ensuring compliance with clinical trial guidelines.

#### Lesson 5: The Need for Detailed Source Material and Human Oversight

In one research protocol, a reviewer found that the human-generated ICF was more accurate in detailing risks and discomforts compared with the LLM-generated version. This discrepancy was evident in the omission of common discomforts associated with COVID-19 tests in the LLM-generated ICF, which occurred because this information was not included in the original research protocol. This underscores a key lesson: “garbage in, garbage out.” For LLMs to produce a comprehensive and accurate ICF, the original research protocol must be thorough and detailed. Furthermore, this finding highlights the importance of having a human-in-the-loop to review and refine the output from LLMs. While LLMs can significantly reduce the effort required to create an ICF—potentially saving up to 90% of the work—the final product still benefits from human oversight. For example, after generating a well-structured ICF key information section with the LLM, researchers can easily tweak the content to better suit their specific audience. One reviewer noted that the LLM-generated ICF had a more technical and clinical tone than the human-generated version. By having researchers to customize the LLM-generated content, the ICF can be tailored to the study audience, while most of the heavy lifting has been accomplished by the LLM.

#### Lesson 6: Ethical and Regulatory Considerations for LLM-Generated Content

With the increasing role of LLMs in generating participant-facing documents, ethical and regulatory concerns must be addressed. Key considerations include ensuring that AI-generated ICFs do not inadvertently introduce bias or misinformation. Furthermore, as LLMs take on more responsibility in clinical settings, regulatory bodies may need to establish guidelines to govern their use. These guidelines could include stipulations on the necessity of human oversight to verify that LLM-generated content is accurate, participant-friendly, and compliant with ethical standards for informed consent.

#### Lesson 7: Techniques for Structured and Accurate Information Extraction

By using effective prompt engineering strategies [31,32] and crafting prompts that were precisely focused on extracting information from the research protocol, we were able to generate a well-structured and neatly formatted output. Key components—such as the introduction, study purpose, procedures, risks and discomforts, benefits, alternatives, cost and compensation, and consent process—were clearly delineated with headers, which improved the organization of the document and made it easier to locate specific information. When crafting the initial prompt to extract key information, we used a combination of techniques:

1. Delimiter usage: Delimiters like ### and [] were used to clearly define boundaries between different sections of the text.
2. Role-playing: Assigning the LLM a specific role, such as “As a Clinical Trial Informed Consent Writer,” provided contextual guidance, resulting in improved performance by making the model’s responses more relevant and focused.

#### Lesson 8: Addressing Practical Benefits and Cost-Savings

Beyond improving the quality of ICFs, LLMs offer the potential to reduce operational costs and administrative burdens in clinical trials. Automating the creation of ICFs can significantly cut down the time spent on document preparation while maintaining compliance with regulatory standards. By quantifying these savings—such as estimating the reduction in hours spent on ICF creation—future studies could further demonstrate the tangible benefits of incorporating LLMs into clinical workflows.

#### Lesson 9: Anticipating Future Advances

As of this publication, GPT-4o mini [33], Mistral Large 2 [34], and Meta Llama 3.1 [35] have been released, each featuring an expanded context window of 128k tokens, making them ideal for these tasks. However, they were not available during the development phase. While 64k tokens are sufficient for handling most clinical research protocols, for more extensive content, these new models would be preferable. That said, the prompts are compatible with all of these models.

### Implications for Practice

The use of LLMs to generate ICFs offers considerable potential for streamlining the informed consent process. By producing more readable, understandable, and actionable content, LLMs can enhance participant comprehension and engagement, potentially improving recruitment and retention in clinical trials. Furthermore, the time savings associated with automated ICF generation can reduce the workload on researchers while ensuring that ICFs remain aligned with regulatory standards for readability and content clarity.

### Limitations and Future Directions

This study’s findings must be interpreted in light of certain limitations. The LLM’s performance is closely tied to the quality of the input research protocol. The LLM’s performance is closely tied to the clarity and completeness of the source material. Ambiguities or inconsistencies in the research protocols can hinder the model’s ability to capture all relevant details to generate accurate and comprehensive ICFs. Future research should focus on improving the clarity of source materials and refining prompt-engineering strategies to optimize LLM performance, particularly in more complex sections such as study procedures.

To address these challenges with procedural details, we used a targeted prompt-engineering approach, which involved having the LLM first summarize the study’s procedures and timeline, and then extract specific details from the summarized text. This method improved accuracy, but ongoing refinement of these strategies is needed to enhance the LLM’s ability to process complex and lengthy sections more effectively.

Another limitation relates to the potential recognizability of LLM-generated text. Although evaluators were blinded to the source of the ICFs and presented with human- and LLM-generated documents in a randomized order, the distinctive textual style of LLM outputs—characterized by active voice, well-organized structure, simplified language, and consistent adherence to readability guidelines—may have inadvertently revealed their origin. This recognizability could introduce subconscious bias into the evaluation process. To address this in future studies, we plan to use text obfuscation techniques, such as paraphrasing or reformatting outputs, to minimize stylistic differences and ensure true blinding. This approach will help strengthen the validity of future comparisons.

Another important limitation of this study is the small sample size, which consisted of only 4 clinical trial protocols. While these protocols provided a useful test bed, the relatively small sample size limits the generalizability of the findings. Future studies should incorporate a broader range of clinical trials from diverse therapeutic areas, phases, and levels of complexity to fully validate the model’s performance. Furthermore, the limited sample size may have contributed to some statistically nonsignificant results, such as those related to procedural details or study alternatives. A larger sample would provide greater statistical power, enabling the detection of smaller but practically significant differences between human- and LLM-generated ICFs.

A larger sample would also better capture the diversity of challenges involved in ICF creation, such as variations in regulatory requirements, medical procedure complexity, and considerations for vulnerable populations. Future research should aim to assess the LLM’s robustness and adaptability across a wider array of clinical trial contexts.

The process of designing and refining prompts, as well as generating the AI-generated ICFs, required a moderate time investment during initial development. However, leveraging the existing prompts and lessons learned in this study would enable future users to complete the process more efficiently and at reduced cost, enhancing the scalability of this approach.

The 0% actionability score for human-generated ICFs reflects a structural issue rather than a methodological flaw. Most institutions do not include explicit actionable sections in the key information portions of their templates. Updating these templates to include actionable instructions would likely improve scores significantly. This highlights a strength of LLM-generated ICFs, which inherently include actionable elements, enhancing the clarity and use of consent forms.

While the LLM demonstrated strong performance, there were instances where it missed details related to the duration and procedural elements of the study. This likely stems from 2 primary challenges: ambiguous or inconsistent presentation of these sections in the research protocols and the verbosity of the text, which can hinder the LLM’s ability to process details efficiently. Our targeted approach of summarizing and then extracting procedural details helped address this issue, but further enhancements are needed to ensure that the LLM can consistently handle such challenges.

Looking ahead, our next goal is to automate the creation of entire ICFs directly from research protocols. This would significantly reduce the time and effort required for ICF development while maintaining consistency and quality. While this study highlights the potential for human oversight to address issues in LLM-generated content, our future research will aim to quantify the time and effort required for revisions to better assess the practical efficiency gains of integrating these models into clinical workflows. We also plan to explore the LLM’s multilingual capabilities to generate ICFs in multiple languages, broadening the recruitment base and promoting diversity and equity in clinical trials. Ensuring that non–English-speaking participants receive ICFs that are as readable and understandable as those in English is crucial for improving inclusivity and representation in research. Future work should also prioritize a thorough examination of ethical concerns, including potential biases in AI-generated content, the need for transparency in AI decision-making processes, and the legal implications of deploying LLMs in clinical trial workflows, to ensure that these tools are implemented responsibly and equitably.

### Conclusions

This study highlights the potential of LLMs to improve the efficiency and quality of ICF generation in clinical trials. While human oversight remains necessary to ensure accuracy in complex sections, and the findings are constrained by the small dataset and evaluation of a single LLM model, LLMs demonstrated potential advantages in producing more readable, understandable, and actionable ICF content. As LLM technology continues to evolve, it holds the promise of further enhancing the informed consent process by facilitating the creation of ICFs that are both participant-friendly and compliant with regulatory standards, thereby improving ethical conduct in clinical research.

## Supplementary material

10.2196/68139 Multimedia Appendix 1 Prompt details.

10.2196/68139 Multimedia Appendix 2 Form for grading scale of informed consent key information sections.

## Abbreviations

AI: artificial intelligence
EHR: electronic health record
ICC: intraclass correlation coefficient
ICF: informed consent form
IRB: institutional review board
LLM: large language model
RUAKI: Readability, Understandability, and Actionability of Key Information
