# Supplementary material for: Transforming Informed Consent Generation Using Large Language Models: Mixed Methods Study
Source: JMIR Med Inform. 2025 Feb 13;13:e68139. doi: 10.2196/68139 (PMC11841745; doi:10.2196/68139)
Supplement: Multimedia Appendix 1 [file medinform-v13-e68139-s001.docx]

Supplementary Material:

Supplementary 1: Chatbot Prompt for Generating Key Information in Informed Consent Forms

As a "Clinical Trial Informed Consent Writer," your task is to write the key information section of the informed consent form using the provided format.

####

Introduction:

We invite you to take part in a research study because you [state reason, such as having a particular medical condition]. Your participation is voluntary, and you are free to say no or to leave the research at any time without any penalties or changes in the quality of healthcare you receive. You will not lose any benefits to which you are otherwise entitled. If you have questions or don’t understand something, please ask.

Purpose of the Study:

The main question this study is trying to answer is [indicate the main purpose(s) of the study in very simple language]. The purpose of this research is to [insert general overview of the purpose and why potential participants are eligible].

Duration and Procedures:

[Please extract the information only from clinical trial research protocol.]

If you decide to join this research study, your participation will last for [insert duration]. During this time, you will need to [provide an overview of study procedures, including number and length of visits, locations, and any specific activities or tests involved, e.g., blood sample collection, physical exams, biopsies, etc.].

Risks and Discomforts:

We will take steps to protect your personal information. However, there is a risk of a breach of confidentiality. There may also be risks that we do not know yet.

[Please extract the information only from Risk and benefit section in the clinical trial research protocol.]

Possible risks and discomforts may include [describe any potential benefits, if applicable].

A detailed description of side effects, risks, and possible discomforts can be found later in this consent form in the section called “What are the risks and possible discomforts from being in this research study?”

Benefits:

[Please extract the information only from Risk and benefit section in the clinical trial research protocol.]

Direct benefits to you are not guaranteed. Potential benefits may include [describe any potential benefits, if applicable].

Your participation may help others in the future by contributing to scientific knowledge about [specific condition].

Alternatives:

You do not have to be in this study to receive care for your [condition]. Other choices include [describe any other options the subject may have]. [If research participants could receive the intervention without being in the research, state: You do not have to be in this study to receive the study drug/device/intervention]. [If there are no alternatives, state: Your alternative is not to take part in the research.]

Cost and compensation:

[Describes any financial payments (or costs) to study participants.]

Consent Process:

[Describes the process by which the reader gives their consent, either by signing a document, verbal agreement, via computer, or other.]

If you are interested in learning more about the study, please continue reading, or have someone read to you, the rest of this document. Take as much time as you need before you make your decision. Ask the study staff questions about anything you do not understand. Once you understand the study, we will ask you if you wish to participate; if so, you will have to sign this form.

#### Clinical Trial Research Protocol:

{ResearchProtocol}

####

The clinical trial research protocol is provided above. Read the research protocol thoroughly and fill in the corresponding informed consent form key information section based on the format provided.

Supplementary 2: Refinement of Content Using RUAKI Indicators

{output_from_supplementary_1}

####

The clinical trial research protocol key information section is provided above. Please ensure it follows the requirements below:

Use active verbs instead of passive verbs.

Avoid scientific jargon (e.g., use "high blood pressure" instead of "hypertension").

Provide a clear definition of the main disease or topic of the study.

Supplementary 3: Optimization for Flesch-Kincaid Grade Level 8 or Below

{output_from_supplementary_2}

####

The clinical trial research protocol key information section is provided above. Please ensure it follows the requirements below:

Readability by Flesch-Kincaid Grade Level should be 8 or below.

Examples of Flesch Reading Grade Level Under 8:

Example 1:

The students eagerly prepared for their science fair, each one hoping to impress the judges with their projects.

Explanation: This sentence is longer with a compound structure, but it still uses relatively simple vocabulary.

Example 2:

The patient was instructed to take the medication twice a day, once in the morning and once at night, to maintain consistent levels in the bloodstream.

Explanation: This sentence provides detailed instructions with a slightly more complex structure but remains accessible.

Example 3:

During the summer, the children spent most of their days at the park, playing games and enjoying the warm weather.

Explanation: The sentence is descriptive with a compound structure, suitable for a grade level of 7-8.

Example 4:

The teacher explained the assignment clearly, ensuring that all the students understood what was expected of them before they began.

Explanation: This sentence includes a dependent clause and more detailed language.

Example 5:

After the meeting, the manager sent out a detailed summary to all team members, highlighting the key points that were discussed.

Explanation: This sentence uses a compound structure with clear, direct language, appropriate for a grade level of 7-8.

Examples of Flesch Reading Grade Level Above 8:

Example 1:

In order to participate in the clinical trial, all eligible candidates must undergo a comprehensive medical evaluation to ensure they meet the stringent criteria set forth by the research team.

Explanation: This sentence is complex, with multiple clauses and advanced vocabulary, suitable for a grade level above 9.

Example 2:

The intricacies of the experimental design required the researchers to meticulously plan every aspect of the study, ensuring that the results would be both valid and reliable.

Explanation: The sentence uses advanced vocabulary and a more complex sentence structure, raising the reading grade level.

Example 3:

While the initial results were promising, further analysis revealed several potential confounding variables that needed to be addressed before drawing any definitive conclusions.

Explanation: This sentence introduces technical terms and a sophisticated structure, appropriate for a higher reading level.

Example 4:

The committee's recommendations were based on a thorough review of the evidence, which included both qualitative and quantitative data collected over several years.

Explanation: The sentence is formal, with more specialized language and a complex sentence structure.

Example 5:

Understanding the potential implications of the data required not only a deep knowledge of the subject matter but also the ability to interpret the results in the context of the broader field of study.

Explanation: This sentence uses advanced vocabulary and a compound-complex sentence structure, making it suitable for a grade level above 9.

Based on the examples provided above, please retain all the information and ensure the content is written at a Flesch-Kincaid Grade Level of 8 or below.

Supplementary 4: Formatting Refinement and Structure Enhancement

{output_from_supplementary_3}

####

The clinical trial research protocol key information section is provided above. Please use the provided content and edit based on the requirement below:

Use markdown headers to clearly label sections or chunks of information. Headers should clearly describe each section to make it easy for readers to scan and find information.

Use 'Keep the sentence 'The purpose of this research is to ...' in Purpose of the Study section.

Uses numbered lists for Risks and Discomforts section.
