# Supplementary material for: Transforming Informed Consent Generation Using Large Language Models: Mixed Methods Study
Source: JMIR Med Inform. 2025 Feb 13;13:e68139. doi: 10.2196/68139 (PMC11841745; doi:10.2196/68139)
Supplement: Multimedia Appendix 2 [file medinform-v13-e68139-s002.docx]

**Supplementary Table 1.** Form for Grading Scale of Informed Consent Key Information Sections

|  | Complete | Partial | Absent | Inaccurate | Comments/observations |
| --- | --- | --- | --- | --- | --- |
|  |  |  |  |  |  |
| Study Purpose |  |  |  |  |  |
| Duration and Procedures |  |  |  |  |  |
| Risks and Discomforts |  |  |  |  |  |
| Benefits |  |  |  |  |  |
| Alternatives |  |  |  |  |  |
| Overall impression |  |  |  |  |  |
